# The Relationship Between Excessive Screen Time, Self‐Harm, and Suicidal Behavior in Adolescents During the COVID‐19 Pandemic: An Integrative Literature Review

**DOI:** 10.1111/jcap.70015

**Published:** 2025-04-17

**Authors:** Mariane Inaraí Alves, Sérgio Alves Dias Junior, Thais Martins, Adriana Olimpia Barbosa Felipe, Patrícia Scotini Freitas, Denis da Silva Moreira

**Affiliations:** ^1^ Universidade Federal de Alfenas Programa de Pós‐graduação em Enfermagem Alfenas Minas Gerais Brazil

**Keywords:** adolescent, COVID‐19 pandemic, internet addiction disorder, mental health, self‐harm behavior

## Abstract

**Introduction:**

Adolescents are being increasingly exposed to digital media, especially in the aftermath of the COVID‐19 pandemic. This reality raises concerns over the effects of this exposure, in addition to an increase in suicidal behavior and self‐harm.

**Aim/Question:**

This study aims to analyze the relationship between excessive screen time, self‐harm, and suicidal behavior in adolescents during the COVID‐19 pandemic.

**Methods:**

This is an integrative literature review. The research question was structured using the PICOT strategy (P—adolescents; I—intensive screen and internet time; O—suicidal behavior and self‐harm; and T—pandemic timeframe). The databases included were: Embase, LILACS, PubMed, Scopus, Cinahl, Web of Science, and Google Scholar. Initially, 1645 studies were found; after reviewing their titles and abstracts, 18 studies met the eligibility criteria.

**Findings:**

The results revealed concerning associations between prolonged exposure and behaviors such as self‐harm and suicidal behavior. Implications for Practice: These findings can assist nurses in identifying young individuals at risk due to inappropriate technology use, enabling the development of tailored interventions, the implementation of educational programs for healthy digital habits, and the promotion of mental well‐being.

**Recommendation:**

In light of the above, measures to mitigate this problem and the risks stemming from prolonged exposure are needed, considering that the adverse effects go beyond the pandemic context.

## Summary

What is known on the subject?

- Adolescence is a period of intense emotions and vulnerability, making it challenging for adolescents to cope with the increasing demands of digital media.
- In this context, the COVID‐19 pandemic has led to drastic changes in young people's routines, resulting in a significant increase in their screen and internet time. This scenario, marked by social isolation and adaptation to new forms of interaction, raises concerns about the impact of intensive technology use on the mental health of this population.
- Recent studies indicate a possible correlation between excessive screen time and an increase in suicidal and self‐harm behaviors among young people. However, there is a gap in the literature regarding this correlation during the COVID‐19 pandemic.

What the paper adds to existing knowledge?

- This study conducted an integrative review of the relationship between excessive screen time, self‐harm, and suicidal behavior in adolescents during the COVID‐19 pandemic.
- The results revealed concerning associations between prolonged screen exposure and behaviors such as self‐harm and suicidal tendencies.

What are the implications for practice?

- These findings can help nurses identify young individuals at risk due to inappropriate technology use, enabling the development of tailored interventions.
- Nurses can implement educational programs for healthy digital habits (Oh et al. 2022), emphasizing awareness of the potential risks of excessive screen time and promoting strategies to balance digital engagement with other activities, such as physical, social, and emotional strengthening activities.
- The results highlight the importance of continuous monitoring of adolescents' mental health and the implementation of measures designed to promote psychological well‐being. To achieve this, it is essential to address their emotional and affective needs through strategies that strengthen socio‐emotional resources, promote resilience, encourage interpersonal bonding, and assist in stress management. These measures enable the creation of an effective support network for adolescents. By integrating these aspects, nurses perform a key role in adopting a holistic approach to adolescent mental health and well‐being.

## 1 Introduction

The frequency of self‐harm among adolescents is a serious concern, which is strongly related to suicide attempts (Hooley et al. 2020) and deaths resulting from suicide. It is estimated that between 40% and 60% of people who complete suicide have engaged in self‐harm behaviors, indicating an increased risk of suicide for these individuals (Geulayov et al. 2019).

Self‐harm behavior involves actions in which an individual intentionally inflicts harm upon themselves (Farkas et al. 2024; Nock et al. 2006). This includes non‐suicidal self‐injury (NSSI), which refers to the deliberate destruction of one's own bodily tissue without the intent to die (American Psychiatric Association 2014; Moutier 2024; Nock et al. 2006), and suicide attempts, which are direct efforts to intentionally end one's own life (Moutier 2024; Nock et al. 2006).

Suicidality encompasses not only suicide attempts but also suicidal ideation, which refers to thoughts about self‐harm, and suicidal planning, which involves formulating a specific plan to complete suicide (Li et al. 2022). In contrast, suicidal behavior refers to a range of actions that include suicide attempts and preparatory behaviors, extending to completed suicide (Moutier 2024).

NSSI typically manifests during early adolescence and can persist for several years (American Psychiatric Association 2014; Moutier 2024). It is important to emphasize that suicide is a significant public health concern worldwide, as it is the third leading cause of death among adolescents (World Health Organization 2021).

In addition, adolescence is a period marked by intense emotions and vulnerability in terms of regulating emotions and behavior, which can hinder the coping ability of adolescents with the growing demands of digital media. Adolescents are increasingly exposed to these media, spending more time chatting online, browsing the internet, and playing electronic games (X. Zhu et al. 2023).

In this context, the COVID‐19 pandemic has led to drastic changes in the routine of adolescents, resulting in a significant increase in their screen, internet time (Resende et al. 2024), smartphone use, and social media (Freitas et al. 2024). This scenario, marked by social isolation and adaptation to new forms of interaction, raises concerns regarding the impact of the intensive use of technology on the mental health of this population (Resende et al. 2024).

The pandemic period was unprecedented, marked by measures such as school closures and lockdowns, which led to physical isolation and triggered fear and uncertainty regarding the future. These factors contributed to increased anxiety, stress, and depressive symptoms (Freitas et al. 2024), in addition to increased loneliness, anxiety, sadness, distancing from friends, and socialization difficulties during the pandemic (Peterle et al. 2022). As a result, many adolescents started to spend several hours on digital media, using virtual interaction as a compensatory mechanism to meet their socio‐emotional needs (Freitas et al. 2024).

In this context, it is noticeable that adolescents are increasingly dependent on mobile devices, a trend that was further intensified by the COVID‐19 pandemic, which increased screen time (Serra et al. 2021). Screen time' refers to time spent with any screen, including smartphones, tablets, television, video games, computers, or wearable technology (Canadian Paediatric Society et al. 2019).

Excessive use of mobile devices can become a problem when it involves loss of control, compulsive use, withdrawal symptoms, and problematic behaviors. This phenomenon, known in the literature as “problematic mobile phone use” or “smartphone addiction,” has a significant negative impact on adolescents' mental health. The condition is typically assessed through questionnaires or by the amount of time spent on the device (H. Kim 2022).

Excessive screen time is defined in the literature in varied ways, generally encompassing exposure instances exceeding 2 (Feng et al. 2024) or 3 h per day (Jin et al. 2022). Recent studies indicate a possible correlation between excessive screen time and an increase in suicidal and self‐harm behaviors among adolescents (Chen et al. 2024). However, there is a gap in the literature regarding this correlation during the COVID‐19 pandemic.

Therefore, this study aims to analyze the relationship between excessive screen time, self‐harm, and suicidal behavior in adolescents during the COVID‐19 pandemic.

## 2 Methods

This is an integrative literature review. This study design enables the search, critical evaluation, and synthesis of the available evidence on the theme being studied. The integrative review method was used with the following stages: development of the research question, sampling or searching the literature for primary studies, data extraction and categorization of the studies, evaluation of the primary studies, interpretation of the results, and presentation of the review (Mendes et al. 2008).

The PICOT strategy was used to develop the research question, with the following criteria: Patient/Population (adolescents), Interest (intensive screen and internet time), Comparator (not applicable), Outcome (occurrence of suicidal behavior and self‐harm), and Time (during the pandemic) (Melnyk and Fineout‐Overholt 2019). In this setting, the guiding question was devised: What is the scientific evidence available on excessive screen time and the occurrence of self‐harm and suicidal behavior in adolescents during the COVID‐19 pandemic?

The inclusion criteria cover primary studies published in Portuguese, English, and Spanish that address suicidal behavior and self‐harm in adolescents (ages 10−19) during the COVID‐19 pandemic (March 11, 2020, to May 5, 2023). Reviews, letters, case studies, and experience reports were excluded.

The databases included Embase, LILACS, PubMed, Scopus, CINAHL, Web of Science, and Google Scholar (gray literature). Health Sciences Descriptors (DeCS) were used in the three languages in LILACS, Medical Subject Headings (MeSH) were used in PubMed, Web of Science and Scopus, and CINAHL Headings, and Emtree Terms were used in Embase, as well as synonyms and keywords.

A single search strategy, adapted for each database, was applied: (“screen time” OR “Mobile Applications” OR Smartphone* OR “Smartphone Addiction” OR “Internet use” OR “Internet Addiction” OR “Internet Addiction Disorder*” OR “Web Use” “Screen Media use” OR “Screen media” OR “Digital media” OR “media addiction” OR “Social Media” OR “Media Addiction, Social” OR “Media Addictions, Social”) AND (Suicide OR “Suicidal Ideation” OR “self injurious behaviour” OR “Self‐inflicted violence” OR “non‐suicidal self‐injury” OR “Nonsuicidal Self Injuries” OR “Intentional Self Injury” OR “Intentional Self Injuries” OR “self harm” OR “Self‐Harm, Deliberate” OR “Self Mutilation” OR “NSSI” OR “Injuries, Self‐Inflicted” OR “Self Mutilation Risk”) AND (adolescent OR adolesc* OR “Adolescent Behavior” OR “Adolescent Health” OR Teenager OR Teenagers) AND (“covid 19” OR “covid‐19” OR “SARS‐CoV‐2” OR “SARS CoV 2” OR “COVID‐19 Pandemic” “COVID 19 Pandemic” OR “COVID‐19 Pandemics” OR “Pandemic, COVID‐19”).

After conducting the search in the databases, the records were exported to the EndNote reference manager, web version (Bramer and Bain 2017), enabling the identification and elimination of duplicate studies. The records were then uploaded to the Rayyan web application (Ouzzani et al. 2016), which was also used to check for duplicate studies. In the first stage, two reviewers analyzed the titles and abstracts of the studies independently and blindly. In the second stage, the selected studies were read in full. In cases of disagreement between the evaluations, a consensus meeting was held with the participation of a third reviewer.

Data extraction was carried out using Google Forms, to collect information on study identification, type of study, objectives, population/sample, evaluation instruments used in the studies, and main results.

The Joanna Briggs Institute (JBI) tools were used to assess the methodological quality of the studies. The JBI offers several specific tools for assessing methodological quality, which are suited to each type of study method (Aromataris et al. 2024). The review protocol was registered on Figshare (10.6084/m9.figshare.27101644) and the adapted guidelines of the Preferred Reporting Items for Systematic Reviews and Meta‐Analyses (PRISMA) were employed (Galvão et al. 2022).

## 3 Findings

**Figure 1:**
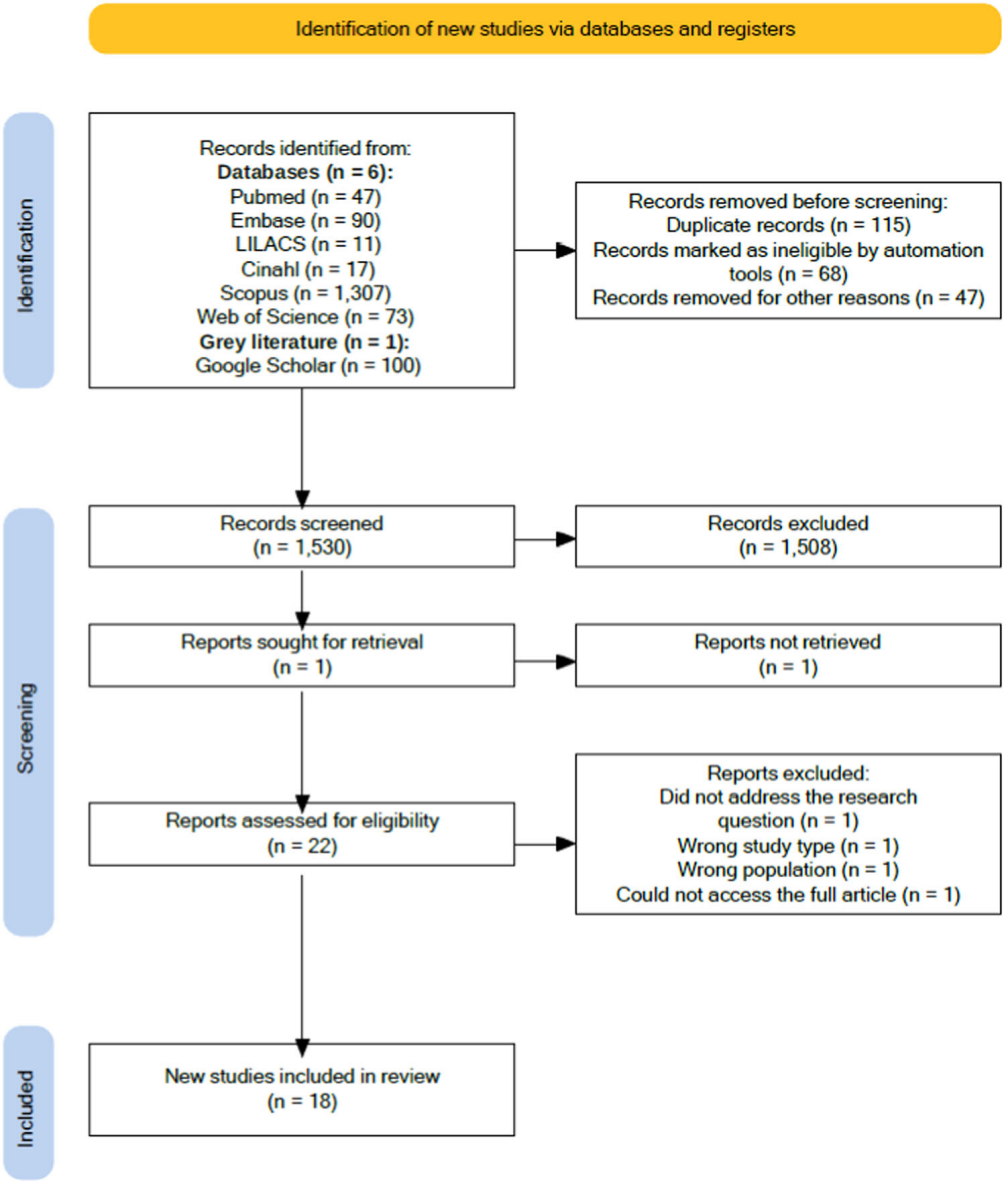
Flowchart of the search and selection process for studies. *Source:* Adapted from PRISMA (Galvão et al. 2022). [Color figure can be viewed at wileyonlinelibrary.com]

The initial search strategy found 1645 studies. After reviewing titles and abstracts and eliminating duplicates, 22 studies were chosen for full analysis. Of these, 18 studies met the eligibility criteria and were included in the synthesis. Figure 1 shows the PRISMA flowchart illustrating the search and selection process for the studies.

These 18 studies included 1,236,385 adolescents aged from 10 to 19. Most studies were conducted in China, with a total of nine studies, followed by South Korea, with six studies. The remaining studies were carried out in the United States, Switzerland, and Taiwan, with only one study in each of these countries.

There is considerable heterogeneity in the instruments used to assess internet addiction, screen time, social media, smartphones, and video games.

Most of the studies included are cross‐sectional descriptive studies (12 studies), five are longitudinal descriptive studies, and one is a case‐control study.

The themes covered in the articles included in the integrative review were organized and analyzed in descending order of frequency. The most recurrent theme was “Mobile phone addictio,” discussed in six studies, followed by “Internet addiction, problematic use, and dependence,” also present in six studies. The “screen time” and “excessive social media time” themes were explored in two studies each, while one study simultaneously addressed “excessive social media time” and “video game addiction.” Most of these studies analyzed the relationship between these themes and suicidal behavior, while only three studies included the self‐harm theme.

The results indicated that during the COVID‐19 pandemic, there was a significant increase in screen time, and this excessive use, along with the adverse effects of the health crisis, emerged as one of the main risk factors for suicidal ideation. Adolescents who spent more than 4 h a day on their smartphones were more likely to experience suicidal ideation, plan, or attempt suicide. Additionally, excessive use of the internet, social media, and video games was associated with self‐harm and suicidal behavior.

Table 1 describes the main characteristics and level of evidence of the studies.

**Table 1:** Main characteristics of the studies included and level of evidence.

Study	Objectives	Method	Population/sample	Assessment instruments	Main results	Level of evidence
Mobile phone addiction and suicidal behavior						
Cha et al. (2023)	To research the association between smartphone use and adverse health outcomes	Descriptive, longitudinal	54,948 adolescents (mean age 15.2 years)	Questionnaire covering health risk behaviors and questionnaire on smartphone use	The number of adolescents in 2020 who spent more time on a smartphone for more than 2 h a day increased from 64.3% in 2017 to 85.7% in 2020 (*p* < 0.001) and 25.5% of adolescents had smartphone addiction in 2020. Negative associations were found between smartphone use time (after 4 h of use) and health outcomes (stress, depressive symptoms, suicidal ideation, and substance use)	VI
Cheng et al. (2024)	To explore the direct effect of mobile phone addiction on suicidal behavior and the indirect effect through poor sleep quality	Descriptive, cross‐sectional	18,900 Chinese teenagers aged from 12 to 18	Questionnaire on suicide ideation, plans, and attempts; mobile phone addiction index (MPAI); Pittsburgh sleep quality index (PSQI); nine‐item patient health questionnaire (PHQ‐9); seven‐item generalized anxiety disorder scale (GAD‐7)	The prevalence of mobile phone addiction and poor sleep quality was 26.2% and 23.1%, respectively. Suicidal behaviors affected 24.4% of participants, with 10.7% ideators, 8.4% planners, and 5.3% attempters. These factors were more prevalent among women living in rural areas. Mobile phone dependence was associated with suicidal ideation (OR = 1.22, IC de 95%: 1.09–1.37, *p* < 0.001) and planning (OR = 1.18, IC de 95%: 1.04–1.34, *p* < 0.05), but not with attempts. Compared to men, women presented a higher prevalence of mobile phone addiction (*p* > 0.001).	VI
Lee and Lee (2023)	To research the prevalence of problematic smartphone use among adolescents and its association with anxiety and suicidal ideation	Cross‐sectional, population‐based	54,948 adolescents with a mean age (15.01, SD: 1.75)	Smartphone overdependence scale; 7‐item generalized anxiety disorder scale; suicidal ideation was assessed using open‐ended questions and a sociodemographic questionnaire	25.1% of adolescents reported experiencing problematic smartphone use (PSU), 33.2% showed symptoms of anxiety, and 10.9% stated that they had seriously considered suicide in the last 12 months. Girls with PSU presented a significantly higher risk of developing anxiety and suicidal ideation compared to girls without PSU (*p* < 0.001). Boys with PSU also presented higher levels of anxiety and suicidal ideation than boys without PSU (*p* < 0.001).	VI
Shinetsetseg et al. (2022)	To explore the associations between smartphone addiction and suicidal ideation as well as Korean adolescents' attempts to recognize the risk of excessive smartphone use	Descriptive, cross‐sectional	41,173 adolescents	It used data from the 2020 Korea youth risk behavior web‐based survey	Adolescents with potentially risky smartphone use presented higher odds of suicidal ideation (OR: 1.50, CI: 1.42−1.60). Similarly, adolescents with high‐risk smartphone use presented a significant risk of suicidal ideation (OR: 2.49, CI: 2.21−2.81) and attempted suicide (OR: 1.87, CI: 1.48−2.27) compared to adolescents who were general users. The girls' scores exceeded the boys' in terms of potential and high risk of excessive smartphone use (*p* < 0.0001).	VI
W. O. Oh and Heo (2024)	To identify the relationships between smartphone addiction, lifestyle risk behaviors, depression, and suicidal behaviors among South Korean adolescents during the COVID‐19 pandemic	Descriptive, cross‐sectional	54,948 young Koreans (mean age = 15.19 years)	It used data from the “2020 Korea Youth Risk Behavior Web‐Based Survey”; smartphone overdependence scale	Smartphone overdependence was positively associated with lifestyle risk behaviors, which in turn were positively associated with suicidal ideation. The direct relationship between smartphone overdependence and suicidal ideation was also statistically significant, suggesting that lifestyle risk behaviors partially mediated the link between smartphone dependence and suicidal ideation. The indirect effect of smartphone overdependence on suicidal ideation was statistically significant. The risk of overdependence, depression, suicidal ideation, and suicide planning was significantly higher among women.	VI
Li et al. (2022)	To research the severity of mobile phone addiction in Chinese teenage students during the COVID‐19 quarantine (T1) and the risk of suicide 5 months later when the pandemic was in remission in China (T2)	Descriptive, longitudinal	1609 students aged 12−18	Demographic and exposure questionnaires related to COVID‐19; telephone dependency index; Chinese daytime sleepiness scale for adolescents; Kutcher adolescent depression scale; suicidal behaviors questionnaire‐revised;	Mobile phone addiction during the quarantine directly predicted suicidality over the next 5 months, even after controlling for the effects of depression and daytime sleepiness. Mobile phone addiction at T1 was significantly related to daytime sleepiness (*r* = 0.316), depression (*r* = 0.312), and suicidality at T2 (*p* < 0.001). Meanwhile, suicidality at T2 was significantly correlated with depression at T2 (*r* = 0.469), and daytime sleepiness at T2 was also significantly correlated with suicidality at T2 (*r* = 0.311). Regression analysis revealed that mobile phone addiction during the COVID‐19 quarantine period could directly predict suicidality regardless of covariates such as mental health and psychosocial factors in the following 5 months (*p* < 0.001).	VI
Internet addiction, problematic use, and dependence, screen time, suicidal behavior, and self‐harm						
Deng et al. (2023)	To research the prevalence of internet addiction (IA) and examine the association between IA, academic satisfaction, and mental health problems in university students	Descriptive, cross‐sectional	22,605 female university students (mean age 19)	Nine‐item patient health questionnaire (PHQ‐9); seven‐item generalized anxiety disorder questionnaire (GAD‐7); insomnia severity index (ISI); internet addiction test (IAT); social support rating scale (SSRS)	66.0% presented internet addiction and were more susceptible to symptoms of depression, anxiety, insomnia, and suicidal ideation. The severity of internet addiction significantly increases mental health problems. In addition, there was a significant interaction between internet addiction and academic satisfaction: participants with high levels of internet addiction had more severe symptoms of depression, anxiety, and insomnia when they had low academic satisfaction (*p* < 0.001).	VI
Yang et al. (2023)	To evaluate the interrelationships between symptoms of Internet addiction and suicidality among primary and secondary school students from the perspective of network analysis	Descriptive, cross‐sectional	5380 elementary and high school students	Internet addiction test (IAT); questionnaire developed by the authors	3161 (58.8%) students reported having symptoms of Internet addiction and 798 (14.8%) reported having suicidal ideation or a suicide plan within the last 2 weeks. Network analysis revealed that IAT16 (“Requesting an extended time to spend online”; node strength = 1.223) was the strongest core symptom in the suicidality‐internet addiction network model, while the suicidality‐IAT4 edge (“Forming new relationships with online users”; edge weight = 0.055) was the strongest edge linking both communities. Symptoms of internet addiction were common among elementary and high school students during the COVID‐19 school lockdown period in China and were significantly associated with suicidality.	IV
Liu et al. (2023)	To develop a new clinical model that could be used as a decision‐making tool to evaluate non‐suicidal self‐harm (NSSI) behaviors in this specific population	Descriptive, cross‐sectional	302 adolescents (aged 10−18)	Demographic variables questionnaire; children's depression inventory (CDI); Pittsburgh sleep quality index questionnaire; the internet addiction test; defeat scale and social avoidance and distress scale	Of the 302 adolescents, 58.9% presented self‐harm behavior. The level of education was significantly associated with adolescents presenting depression (*p* = 0.02), self‐harm, low self‐esteem (*p* < 0.001) on the CDI, and sleep disorders (*p* = 0.02). Internet addiction contributed independently to the emergence of self‐harm in adolescents with depression in a statistically significant way (*p* < 0.01). The scores on the low self‐esteem factor of the CDI, scores on the Internet addiction test, and scores on sleep disorders had a high assessment efficiency for the presence of self‐harm in adolescents with depressive disorders. Self‐harm was accurately identified by having a family history of psychosis, a behavioral disorder, biological factors, and clinical characteristics, which included low self‐esteem, sleep disorders, and Internet use.	VI
Tang et al. (2024)	To identify whether depression mediated the association between problematic internet use and non‐suicidal self‐harm in a large sample of Taiwanese adolescents during the COVID‐19 outbreak	Descriptive, cross‐sectional	1060 students from secondary schools (mean age = 14.66 years)	Chinese internet addiction scale (CIAS‐R); depression anxiety and stress scale (DASS); depression subscale; non‐suicidal self‐harm (developed by the authors)	The results showed that the mean indirect effect of problematic Internet use on non‐suicidal self‐harm due to depression was statistically significant (*p* < 0.001). However, the mean direct effect of problematic Internet use on self‐harm was not significant (*p* = 0.134). Prevalence of non‐suicidal self‐harm in the sample: 40.75%	VI
Xiong et al. (2023)	To analyze whether life satisfaction can mitigate the longitudinal associations between problematic Internet use and deliberate self‐harm	Descriptive, longitudinal	6092 Chinese adolescents (mean age = 10.32 years)	Young internet addiction test; satisfaction with life scale; deliberate self‐harm inventory nine‐item version	Associations were found between problematic Internet use and life satisfaction (*p* > 0.001), and life satisfaction and self‐harm (*p* > 0.001). More severe problematic internet use was positively related to higher levels of self‐harm in T1 (*ÿ* = 0.35, *p* < 0.001), T2 (*ÿ* = 0.24, *p* < 0.001), and T3 (*ÿ* = 0.25, *p* < 0.001), respectively. The mean prevalence of non‐suicidal self‐harm was 4.90%.	IV
J. Oh, Kim, et al. (2024)	To compare screen time during the pandemic and its long‐term trend	Descriptive, longitudinal	913,191 Korean adolescents (mean age = 15.1 years)	Korea youth risk behavior web‐based survey questionnaire (national survey)	Regarding the psychological aspect, increased sadness, and suicidal thoughts were associated with increased screen time before and during the pandemic (*p* < 0.001). Sadness (*β* diff, 0.018; IC 95%, 0.007–0.029) and suicidal thoughts (*β* diff, 0.028; IC 95%, 0.016–0.039) contributed to the vulnerability of screen exposure during the pandemic.	VI
Zhang et al. (2024)	To examine the mediating roles of insomnia, nightmares, and social jetlag in the association of problematic Internet use with suicidal ideation in adolescents	Descriptive, cross‐sectional	39,731 adolescents (mean age = 13.49 years)	Patient health questionnaire‐9; Chinese internet addiction scale‐revised (CIAS‐R); questionnaire developed by the authors	The general prevalence of suicidal ideation was 18.6%, with 14.4% for men and 23.6% for women. Suicidal ideation was significantly associated with problematic internet use (OR = 1.93, 95% CI: 1.80−2.10), insomnia symptoms (OR = 2.00, 95% CI: 1.84−2.17), frequent nightmares (OR = 1.98, 95% CI: 1.84−2.12), and social jetlag (OR = 1.31, 95% CI: 1.19−1.45) in the total samples. The direct effects of problematic internet use on suicidal ideation were 1.95 (95% CI: 1.75−2.18) and 1.91 (95% CI: 1.69−2.13) for men and women, respectively.	IV
Jin et al. (2022)	To research the association between screen time and fast food intake and suicidal behavior in Chinese adolescents	Descriptive, cross‐sectional	16,853 participants, with a mean age of 14.71 years (SD = 1.76)	The study measured screen time based on two open‐ended questions regarding the daily use of devices for games, videos, and TV during the week and at weekends, classifying it as high screen time when over 2 h per day were spent. Fast food intake was assessed by weekly frequency, and suicidal behaviors (ideation, plans, and attempts) were analyzed with binary questions (“yes” or “no”) referring to the last year. Psychological symptoms were measured using the brief instrument on psychological health of youths, and sociodemographic data was collected using a questionnaire	Approximately 15.8% of the sample reported screen time > 2 h per day during the week, while 57.3% reported screen time > 2 h per day at weekends. The prevalence of fast food intake was 48.2%. There were positive correlations between screen time, fast food intake, and suicidal behaviors. Interaction analyses showed that increased screen time on weekdays and fast food intake were related to suicidal ideation (*p* < 0.001). Similarly, increased screen time on weekends and fast food intake were also associated with suicidal ideation (*p* < 0.001). Similar additive interaction effects were found between increased screen time and fast food intake in relation to suicidal planning. Positive additive interaction effects were found for increased screen time during weekdays and fast food intake in relation to suicide attempts (*p* < 0.001).	VI
Feng et al. (2024)	To explore the associations between screen time, cyberbullying victimization, and suicide during the COVID‐19 pandemic in high schools for females and for males	Descriptive, cross‐sectional	3982 participants (women: 49%; men: 51%; age 15−17: 74%)	The related questions in the youth risk behavior survey 2021 (YRBS) data set on suicidal behavior; the question in the survey regarding screen time (asked about the hours spent) on video games and social media; the question regarding cyberbullying in the YRBS survey was “During the past 12 months, have you experienced electronic bullying?”; demographic questionnaire: age, race, and biological sex	Experiencing a high screen time increased the odds of suicidal ideation (men: OR 1.50; women: OR 1.47), suicide plans (men: OR 1.56; women: OR 1.45), and suicide attempts (women: OR 1.23). Cyberbullying significantly increased the odds of suicidal ideation (women: OR 3.69; men: OR 4.50), suicide plans (women: OR 3.74; men: OR 5.03), and suicide attempts (women: OR 4.24; men: OR 4.70). The differences between the sexes were evident, with more pronounced effects in women in relation to the mediation of impacts on suicidal ideation (*p* < 0.001), suicide attempts (*p* < 0.001), and suicide in general (*p* < 0.01).	VI
Social media and video games, suicidal behavior, and self‐harm						
J. Zhu et al. (2022)	To describe the behavioral characteristics of non‐suicidal self‐harm in adolescents and to analyze the factors that influence the addictive characteristics of non‐suicidal self‐harm behavior	Case‐control study	Case group: 84 adolescents who reported non‐suicidal self‐harm (mean age = 15.37 years); control group: 84 healthy adolescents (mean age = 14.89 years).	Children's memories scale (CMS); perceived social support scale (PSSS); perceived stress scale (PSS); Bergen social media addiction scale (BSMAS); video game dependence scale (VDG‐S)	The BSMAS and VDG‐S scores of the case study were significantly higher than those of the controls (*t* = 2.27, *p* = 0.01; *t* = 2.44, *p* = 0.02, respectively). The risk factors for non‐suicidal self‐harm traits were as follows: being female, an only child, scoring high on the video game addiction scale, scoring high on excessive interference in the father's parenting style, and scoring high on punishments and excessive interference in the mother's parenting style.	IV
Dumont et al. (2024)	To research the prevalence and risk factors for suicidal ideation in adolescents via a population‐based sample from Switzerland, 2 years after the onset of the pandemic	Descriptive, longitudinal	492 teenagers (aged 14−17)	Suicidal ideation was assessed using open‐ended questions covering suicidal ideation, planning, and attempts. Santé‐Québec psychological distress index; Rosenberg self‐esteem scale; Bergen social media addiction scale; bullying and cyberbullying and academic difficulties were assessed via self‐report	In general, 71 (14.4%) adolescents reported having experienced suicidal ideation within the last 12 months. The results showed that girls were significantly more likely to experience suicidal ideation, as were adolescents who identified as lesbian, gay, or bisexual, or who reported experiencing severe psychological distress, low self‐esteem, low social support, academic difficulties, or suffering from bullying. In addition, adolescents who reported excessive social media time, smoking, alcohol consumption, or experiencing a severe impact from the pandemic had a higher prevalence of suicidal ideation. High psychological distress, low self‐esteem, identifying as lesbian, gay, or bisexual, suffering from bullying, extensive screen time, and a severe impact from the COVID‐19 pandemic were the main risk factors for suicidal ideation. In addition, there was a moderate association between the negative impact of the COVID‐19 pandemic and suicidal ideation.	VI
D. Kim et al. (2024)	To research the similarities and differences in risk factors for suicide among adult and adolescent women in South Korea and to identify subtypes of suicidal ideation or suicide attempts in each group	Descriptive, cross‐sectional	495 adolescent women aged from 13 to 18 and 480 adult women aged from 20 to 39.	The study used several questionnaires to assess psychological, behavioral, and social aspects: Mental disorders and psychiatric symptoms: PDS‐5 and CPSS‐V scales for adults and adolescents, respectively. Depression: CES‐D and CES‐DC scales. Alcohol abuse disorder: CAGE3 scale. Suicide ideation or attempt: C‐SSRS scale. Internet use: Korea Press Foundation questionnaire. Problematic internet use: GPIU2 scale. Online behavior: Questionnaire by Ybarra et al. Communication and social networks: Questionnaire based on Kim's research. Social support in networks: ISEL‐12 scale. Social media use characteristics: Specific questionnaire. Self‐evaluation and positive presentation: Arbel and Kim's scale. Narcissism: Hollenbaugh's questionnaire. Strategies for appreciation on social networks: Dominick's questionnaire. Distress in social networks: Kahn's questionnaire	The risk factors for adolescent females were linked to interpersonal experiences and relationship needs. Two subtypes of adolescents with suicidal behavior were identified: the first was characterized by intense psychiatric symptoms, adverse childhood events, and a strong social connection network; the second was marked by frequent use of social networks, high online sexual victimization, and a sense of social security.	VI

*Source:* Prepared by the authors.

Tables 2 and 3 describe the assessment of the methodological quality of the studies included based on the JBI critical appraisal checklist for cross‐sectional analytical and case‐control studies (Aromataris et al. 2024). In general, the studies presented good methodological quality, meeting most of the criteria assessed.

**Table 2:** Assessment of the methodological quality of studies based on the JBI for analytical cross‐sectional studies.

Estudo	Q1	Q2	Q3	Q4	Q5	Q6	Q7	Q8
Cha et al. (2023)	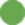	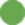	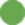	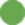	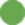	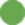	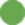	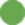
Cheng et al. (2024)	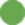	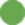	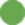	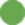	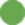	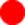	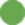	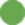
Deng et al. (2023)	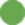	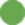	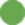	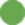	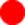	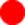	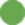	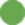
Dumont et al. (2024)	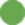	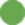	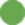	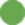	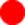	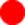	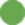	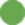
Feng et al. (2024)	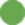	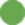	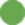	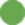	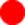	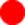	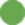	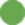
Jin et al. (2022)	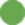	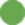	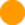	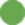	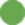	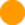	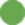	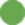
D. Kim et al. (2024)	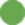	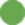	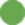	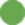	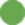	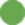	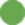	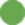
Lee and Lee (2023)	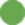	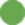	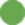	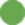	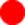	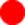	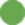	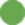
Li et al. (2022)	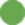	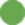	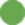	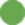	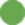	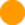	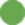	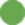
Liu et al. (2023)	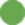	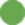	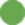	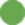	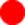	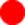	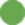	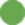
J. Oh, Kim, et al. (2024)	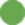	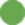	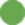	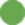	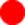	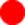	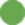	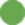
W. O. Oh and Heo (2024)	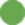	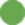	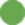	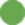	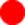	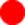	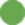	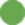
Shinetsetseg et al. (2022)	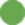	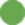	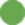	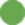	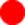	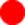	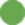	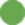
Tang et al. (2024)	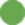	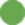	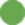	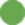	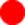	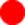	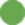	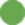
Xiong et al. (2023)	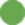	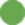	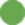	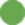	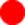	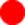	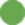	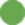
Yang et al. (2023)	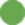	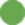	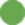	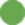	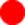	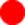	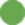	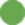
Zhang et al. (2024)	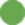		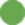	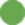	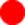	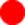	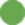	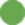

*Source:* Prepared by the authors.

Key: Yes: 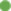 No: 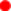 Unclear: 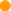

**Table 3:** Assessment of the methodological quality of studies based on the JBI for case‐control studies.

Estudo (Caso‐Controle)	Q1	Q2	Q3	Q4	Q5	Q6	Q7	Q8	Q9	Q10
J. Zhu et al. (2022)	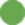	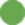	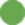	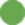	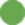	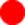	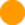	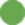	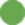	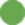

*Source:* Prepared by the authors.

Key: Yes: 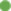 No: 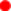 Unclear: 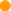

After analyzing the studies collected, they were organized into four categories: Mobile phone addiction and suicidal behavior; internet addiction, problematic use, and dependence, screen time, suicidal behavior, and self‐harm; social media, suicidal behavior, and self‐harm; and impact of screen time, smartphone use, and the internet on the emotional and behavioral health of adolescents.

## 4 Discussion

### 4.1 Mobile Phone Addiction and Suicidal Behavior

Three studies analyzed the prevalence of smartphone addiction among teenagers, revealing a mean value of 25.6% (Cha et al. 2023; Cheng et al. 2024; Lee and Lee 2023). One of the studies found that 22.1% of adolescents were considered to belong to a potential risk group, while 3.0% were classified as high‐risk smartphone addiction users (Shinetsetseg et al. 2022).

There was an increase in the rate of adolescents using their mobile phones for more than 2 h a day during the pandemic, compared to 2017. In addition, using the device for more than 4 h was associated with worse health conditions, which included suicidal ideation (Cha et al. 2023). Smartphone dependence and addiction have been related to suicidal ideation (Cha et al. 2023; Cheng et al. 2024; Oh and Heo 2024; Shinetsetseg et al. 2022), suicide planning (Cheng et al. 2024), and suicide attempts (Shinetsetseg et al. 2022).

In terms of sex, female adolescents were more likely to develop mobile phone addiction (Cheng et al. 2024; W. O. Oh and Heo 2024; Shinetsetseg et al. 2022).

Furthermore, it was found that smartphone addiction during the COVID‐19 quarantine period could predict suicidality (suicidal ideation, suicidal plan, and suicide attempts), both directly and indirectly, 5 months after the onset of the pandemic, when it was in remission (Li et al. 2022).

Although the results of this study indicate a negative impact of smartphone use, it is important to consider research that suggests opposite findings.

An exploratory study reveals that, overall, smartphone use is associated with modest but positive increases in well‐being, especially after prolonged periods of use. Participants often reported higher levels of well‐being after intense use, suggesting that smartphone‐mediated interactions can provide emotional and social rewards, creating a cycle of positive reinforcement. While these effects may be temporary, they can accumulate and contribute to well‐being (Marciano et al. 2022).

Another study conducted by Rudolf and Kim (2024) identified that social and active smartphone use, such as communication with friends and family, predicts fewer depressive symptoms (Rudolf and Kim 2024).

Furthermore, there is no evidence supporting the effectiveness of current school policies that prohibit cellphone use during school hours. This situation indicates the need for improvement in these approaches and further research on the subject (Goodyear et al. 2025).

Therefore, the interaction between smartphone use and well‐being is complex and should be analyzed considering individual experiences, as each adolescent reacts uniquely to these technologies (Marciano et al. 2022).

### 4.2 Internet Addiction, Problematic Use, and Dependence, Screen Time, Suicidal Behavior, and Self‐Harm

Symptoms of internet addiction, such as escapism (excessive use to avoid real‐life problems or emotions), compulsivity (difficulty controlling online use despite negative consequences), and dependency (an overwhelming need to be online, with withdrawal symptoms when unable to access the internet), became more prevalent during the COVID‐19 school lockdown period. These symptoms have been significantly related to suicidality (Yang et al. 2023).

The mean prevalence of internet addiction among adolescents, as found in two studies, was 62.4% (Deng et al. 2023; Yang et al. 2023). Internet use was a significant risk factor for suicidal self‐harm in depressed adolescents during the COVID‐19 pandemic (Liu et al. 2023). Those who engaged in problematic Internet use (PIU) were more likely to suffer from depression, which exerts a complete mediating role between PIU and the practice of non‐suicidal self‐harm (Tang et al. 2024).

PIU was also emphatically associated with self‐harm (Xiong et al. 2023) and suicidal ideation (Zhang et al. 2024). Spending more time online and having online relationships were the two items evaluated that were most closely related to suicidality (Yang et al. 2023).

Screen time has also increased during the COVID‐19 pandemic, which has contributed to an increased vulnerability to prolonged screen exposure (J. Oh, Kim, et al. 2024). One study considered spending 3 h or more a day as excessive (Jin et al. 2022), while another set the threshold at more than 2 h (Feng et al. 2024).

The COVID‐19 pandemic has negatively impacted the lives of adolescents, with excessive screen time and the adverse effects of the health crisis being the main risk factors for suicidal ideation (Feng et al. 2024). These factors have also contributed to an increased probability of suicidal ideation (Feng et al. 2024; Yang et al. 2023; Zhang et al. 2024), with stronger correlations found in women (Feng et al. 2024). There are also associations with suicide planning and attempts (Jin et al. 2022). It was found that increased sadness and suicidal thoughts were associated with increased screen time before and during the pandemic (J. Oh, Kim, et al. 2024).

On the other hand, research indicates that when used in moderation, for less than 2 h a day, screen activities can bring benefits to mental health and well‐being, as well as positively influence other areas related to health and behavior, such as sleep, physical activity levels, classroom conduct, and academic performance (Sanders et al. 2024; Przybylski and Weinstein 2017; Przybylski et al. 2020).

Health professionals can guide families in promoting balanced screen and digital media use by following four key principles. First, it is essential to manage screen time by creating family plans, monitoring use, and setting limits. It is also advisable to avoid media multitasking and maintain open dialogue about online safety. Next, encouraging meaningful screen use is important, prioritizing face‐to‐face interactions and educational content. Additionally, parents should model healthy habits by reducing excessive screen use and promoting screen‐free moments. Finally, it is important to monitor signs of problematic use, such as irritability or interference with sleep and social relationships, to help prevent negative impacts (Canadian Paediatric Society et al. 2019).

### 4.3 Social Media and Video Games, Suicidal Behavior, and Self‐Harm

A significant risk factor for non‐suicidal self‐harm is video game addiction. In addition, adolescents with non‐suicidal self‐harm scored higher on excessive use of social media and video game addiction (J. Zhu et al. 2022).

The prevalence of suicidal ideation and suicide attempts was higher in girls than in boys (Dumont et al. 2024). Frequent use of social networks and high online sexual victimization were associated with suicide in female adolescents (D. Kim et al. 2024).

Despite the negative effects identified in this study, there are also benefits associated with digital engagement. Socializing through social media, recreation, and learning through video games can offer significant advantages for adolescents in various areas. Among the observed benefits, improvements in cognitive functions such as executive control, memory, and problem‐solving stand out, as well as the strengthening of socialization, creativity, and emotional support. Technology can also promote greater autonomy, competence, and well‐being (Haddock et al. 2022).

While the World Health Organization highlights the risks of excessive social media use, it also acknowledges the benefits of responsible use. Adolescents who frequently engage with social media but without negative effects reported greater social support from their peers and stronger social connections (World Health Organization 2024).

### 4.4 The Impact of Screen Time, Smartphone Use, and the Internet on the Emotional and Behavioral Health of Adolescents

Excessive smartphone use, particularly for more than 4 h a day, has been associated with various behavioral and emotional issues. Studies indicate that this practice can lead to increased stress and dissatisfaction with sleep quality (Cha et al. 2023), in addition to being related to depressive symptoms (Cha et al. 2023; Li et al. 2022) and anxiety disorders (Lee and Lee 2023).

Furthermore, there is a significant relationship between excessive dependence on mobile devices and increased alcohol consumption, higher obesity rates (Cha et al. 2023), impaired sleep quality (Cheng et al. 2024), and daytime sleepiness (Li et al. 2022). Among adolescents, smartphone dependence can result in social isolation and deterioration of interpersonal relationships, which may ultimately lead to loneliness and mental breakdown (Shinetsetseg et al. 2022). These findings suggest that prolonged smartphone use promotes harmful behaviors and has adverse repercussions on both physical and mental health (Cha et al. 2023).

Internet addiction has also been widely associated with emotional and behavioral disorders. Research indicates its relationship with depression (Deng et al. 2023; Tang et al. 2024; Liu et al. 2023), anxiety, and sleep problems. Additionally, individuals exhibiting this behavior report lower levels of social support, a higher prevalence of smoking and alcohol consumption, a family history of psychosis, previous psychotic disorders, lower academic progress rates, and higher levels of academic dissatisfaction (Deng et al. 2023).

Adolescents with PIU have reported a higher frequency of sleep disturbances, including insomnia and frequent nightmares (Zhang et al. 2024), as well as lower life satisfaction (Xiong et al. 2023). PIU is also associated with social jetlag, characterized by misalignment between sleep schedules on school days and weekends, making it difficult to maintain a regular sleep routine. According to Yang et al. (2023), internet addiction can lead to symptoms such as depression, irritability, and nervousness, which were reported by adolescents exclusively when they were offline. Additionally, there is a perception of sleep loss due to late‐night internet access (Yang et al. 2023).

Increased screen time before and during the pandemic has also been related to higher levels of sadness. Social factors such as smoking, alcohol consumption, low parental education levels, reduced socioeconomic status, and a lower frequency of physical activity have also been associated with increased screen time, with these associations becoming more pronounced during the pandemic (J. Oh, Kim, et al. 2024). Moreover, screen time is consistently correlated with unhealthy eating habits (Jin et al. 2022).

Another concerning effect of excessive screen time is the increased vulnerability to cyberbullying. Elevated screen use (more than 3 h daily) significantly increased the odds of cyberbullying victimization among female adolescents (Feng et al. 2024). Similarly, frequent social media use among adolescents has been associated with online sexual victimization (D. Kim et al. 2024), indicating the psychological and social negative impacts related to excessive internet use.

This review was limited regarding the restricted use of databases and the use of alternative terms in the search strategy.

Despite this limitation, this is a pioneering study in terms of investigating the negative relationship between excessive screen use during the COVID‐19 pandemic, self‐harm, and suicidal behavior.

Given the growing concerns regarding the impact of excessive screen time on adolescent mental health, especially its association with self‐harming and suicidal behaviors during the COVID‐19 pandemic, it is essential to implement effective mental health interventions in clinical and school settings. Nursing care for adolescents should include a diverse approach, encompassing physical activities (Teixeira et al. 2020), health education initiatives (Oh et al. 2022), therapeutic groups, cognitive‐behavioral therapy, harm reduction strategies, motivational interviewing, individual consultations, as well as the development of healthy interpersonal relationships and a systematic care plan. Integrating families, peers, and the school environment into these interventions is crucial to ensure a holistic and effective approach to adolescent mental health care (Teixeira et al. 2020).

Integrative community therapy (ICT), an integrative and complementary practice, can be an essential tool in addressing these challenges by providing a structured and supportive space for adolescents to express their emotions, develop coping strategies, and strengthen social connections. As an integrative and complementary health practice, ICT has been shown to reduce symptoms of anxiety and depression while reinforcing emotional and social support networks. Therefore, it is recommended that nursing professionals should incorporate ICT into their interventions as a strategy to promote mental well‐being and mitigate the negative effects of excessive screen time on adolescents (Alves, Felipe, Bressan, et al. 2021; Alves, Felipe, and Moreira 2021; Felipe, Alves, De Andrade, Resck, et al. 2023; Felipe, Alves, de Andrade, Bressan, et al. 2023).

## 5 Conclusion

The COVID‐19 pandemic has directly affected the way we communicate, online time has increased significantly, and the use of social media and the internet has become increasingly frequent and common. We have witnessed the adverse effects of the significant increase in exposure to screens, the use of social media, the internet, and video games on adolescents' mental health. This scenario has led to adverse impacts, with evidence of concerning associations between prolonged exposure and behaviors such as self‐harm and suicidal ideation.

In light of this, there is a need for measures designed to mitigate this problem and the risks stemming from prolonged exposure, considering that the adverse effects go beyond the pandemic setting.

Measures targeting the mental health of adolescents, who are a vulnerable population and are increasingly exposed to social media and the internet, are needed, along with the risks of this exposure. Prevention and promotion measures are recommended in schools, which is where they spend most of their time. These measures should include encouraging activities that promote a balance between the use of technology and well‐being, as well as the adoption of healthy digital habits, strengthening resilience and reducing the risks associated with prolonged exposure.

To effectively address these challenges, it is essential to adopt concrete strategies that promote the mental well‐being of adolescents. In this regard, a holistic care model that combines physical activities, health education, and emotional support can be crucial for improving the mental health of this population, especially considering the challenges posed by excessive technology use. The integration of these approaches provides more comprehensive care, helping to build emotional resilience, strengthen coping skills, and balance technology use with adolescents' general well‐being.

Nursing professionals perform a crucial role in this process. By offering comprehensive support, promoting a sense of belonging, encouraging emotional expression, and developing coping strategies to manage stress and anxiety, they ensure that adolescents receive the necessary care. Additionally, interventions should include families, peers, and the school community, creating a strong support network. By incorporating these practices into nursing care, it is possible to address concerns regarding adolescent mental health and provide an effective response to the challenges they face.

## Author Contributions

Mariane Inaraí Alves and Sérgio Alves Dias Junior were responsible for conceptualization, methodology, formal analysis, writing and review and editing. Patrícia Scotini Freitas was responsible for methodology and review and editing. Thais Martins and Adriana Olimpia Barbosa Felipe were responsible for conceptualization, methodology, writing and review and editing. Denis da Silva Moreira was responsible for review and editing. All authors have read and approved the final version submitted and assume public responsibility for all aspects of the work.

## Conflicts of Interest

The authors declare no conflicts of interest.

## Data Availability

Data sets related to this article will be available upon request to the corresponding author.
